# Case report: an unexpected link between partial deletion of the *SHANK3* gene and Heller’s *dementia infantilis,* a rare subtype of autism spectrum disorder

**DOI:** 10.1186/s12888-015-0631-6

**Published:** 2015-10-21

**Authors:** Anne Philippe, Yann Craus, Marlène Rio, Nadia Bahi-Buisson, Nathalie Boddaert, Valérie Malan, Jean-Paul Bonnefont, Laurence Robel

**Affiliations:** Paris Descartes University, Sorbonne Paris Cité, Institut Imagine, UMR1163, Paris, France; Department of Child and Adolescent Psychiatry, APHP Hôpital Necker Enfants Malades, Paris, France; Pediatric Neurology, APHP Hôpital Necker Enfants Malades, Paris Descartes University, Sorbonne Paris Cité, Institut Imagine, Paris, France; Department of Imagery, APHP Hôpital Necker Enfants Malades, Paris, France; Department of Cytogenetic, APHP Hôpital Necker Enfants Malades, Paris, France; Molecular Genetics Unit, APHP Hôpital Necker Enfants Malades, Paris, France; CESP, INSERM U1178, Univ., Paris-Descartes, USPC, Paris, 75014 France; APHP Hôpital Necker Enfants Malades, Paris, France

**Keywords:** *SHANK3*, 22q13.3 deletion syndrome, Autism, ASD, Childhood disintegrative disorder (CDD), Heller syndrome, Regression

## Abstract

**Background:**

Deletions and mutations involving the *SHANK3* gene lead to a nonspecific clinical presentation with moderate to profound intellectual disability, severely delayed or absent speech, and autism spectrum disorders (ASD).

Better knowledge of the clinical spectrum of *SHANK3* haploinsufficiency is useful to facilitate clinical care monitoring and to guide molecular diagnosis, essential for genetic counselling.

**Case presentation:**

Here, we report a detailed clinical description of a 10-year-old girl carrying a pathogenic interstitial 22q13.3 deletion encompassing only the first 17 exons of *SHANK3*.

The clinical features displayed by the girl strongly suggested the diagnosis of *dementia infantilis,* described by Heller in 1908, also known as childhood disintegrative disorder.

**Conclusion:**

Our present case confirms several observations according to which regression may be part of the clinical phenotype of *SHANK3* haploinsufficiency. Therefore, we think it is crucial to look for mutations in the gene *SHANK3* in patients diagnosed for childhood disintegrative disorder or any developmental disorder with a regressive pattern involving social and communicative skills as well as cognitive and instinctual functions, with onset around 3 years.

## Background

Deletions and mutations in the *SHANK3* gene have been repeatedly found when screening cohorts of patients assessed for autism spectrum disorder [1–3], for pervasive developmental disorder not otherwise specified (PDD-NOS) [4], for schizophrenia [5], for non-syndromic intellectual disability [6] and for symptomatic continuous spike and waves during slow-wave sleep syndrome [7].

Patients with *SHANK3* haploinsufficiency exhibit an unspecific clinical profile with moderate to profound intellectual disability, severely delayed or absent speech, hypotonia, and autism spectrum traits [1–3].

The *SHANK3* gene codes for a scaffold protein in the postsynaptic densities (PSD) of excitatory glutamatergic synapses in the central and peripheral nervous system [1]. The protein complex organized by *SHANK3* performs different functions in the postsynaptic membrane including actin-based cytoskeletal remodelling, synapse formation, AMPA receptor endocytosis, and regulation of synaptic transmission and plasticity. *SHANK3* mutant mice show a loss of spines, a reduction in spine volume, and decreased PSD thickness in the adult, suggesting a phenotype of reduced or delayed synapse maturation [8].

Here, we report a detailed clinical description of a 10-year-old girl presenting with a short interstitial 22q13.3 deletion encompassing only the first 17 exons of the *SHANK3* gene, similar to that of another patient (arr 22q13.33 (51,080,647–51,137,385)x1 dn, hg19) and previously reported as pathogenic in Bonaglia et al. (Patient 37) and in Figura et al. (Patient 4) [9, 10].

Our objective was to improve knowledge about the developmental course of *SHANK3* haploinsufficiency in order to facilitate clinical care monitoring and to help in molecular diagnosis, essential for genetic counselling. Indeed, novel *SHANK3* variants are more and more often detected in individuals with unexplained developmental delay or ASD, as a result of the increasing use of next-generation sequencing in routine genetic diagnosis (syndrome-targeted panel, whole exome or whole genome sequencing) [11, 12]. However, interpretation of these novel *SHANK3* variants is challenging. Pathogenicity assessment is based on family co-segregation, functional predictions, the degree of conservation of amino-acids and also on the analysis of precise phenotypical descriptions [1, 13, 14].

Regression may be an important feature of *SHANK3* haploinsufficiency, as suggested by recent case reports pinpointing behavioural regression during the life course of patients with an unstable mood disorder, together with a progressive loss of skills during adolescence [15, 16] or in adulthood [17, 18].

Here, we report for the first time the case of a 10-year-old girl presenting a childhood disintegrative disorder also known as Heller’s *dementia infantilis,* associated with a deletion in the *SHANK3* gene.

## Case presentation

L. is the second child of first-degree-related healthy parents born in Algeria. Her parents separated when she was 18 months old and she has had no further contact with her father since she was 6. She was born through vaginal birth at full term (BW = 3.570 kg; BS = 51.5 cm; HC = 35 cm; Apgar = 10/10). She experienced a slowing of the embryonic cardiac rhythm during delivery, and recovered quickly. According to her mother, L.’s psychomotor and social development were in the normal range until 2 years of age; she did not notice any difference compared to the early infancy of her son, aged 10: good early interactions, appropriate eye contact, development of appropriate imitation, babbling, shared attention skills and use of functional objects. She was able to sit when she was 6 months old, started to walk at 12 months. Babbling was present before 12 months; she was able to duplicate syllables at 9 months, to pronounce her first words at 10 months (daddy, nanny), and first associations of words around 18 months. When she was 2 years old, she could use a few words appropriately (bread, cookies, bread, hello, thanks, more, the names of her brother and sister). However, progress in language was slow, and L. never produced any sentence. Toilet-training was acquired by day and night around 2 years of age, and lost gradually from the age of 5.

L. came to the attention of the school teacher as soon as she started to go to school, when she was in her fourth year, because she was not progressing. Her language was very poor, and she had difficulty focusing her attention. She started to see a child psychiatrist, a psychologist and a psychomotor therapist. Despite these therapies, she became more and more agitated during the second and the third year of school, so that she needed a school assistant. In the same period L. became socially withdrawn: she no longer played with her peers, did not follow the teacher’s instructions either. At home, her activities became more impoverished. She enjoyed making noises with car games, and liked undressing her dolls. Between 5 and 6 years of age, L. experienced three sudden episodes at three different times when she had fever, characterized by extreme fear, followed by a fall onto her knees without loss of consciousness. These episodes were interpreted as febrile seizures, and were treated with sodium valproate after the third episode, although no electroencephalogram (EEG) was available during the crisis, and no abnormality was detected on EEGs between the episodes. According to her mother, after these episodes, L.’s state worsened, with loss of language skills, loss of toilet-training, and aggravation of her agitation. Monotherapy with methylphenidate was introduced to decrease the agitation but caused tolerance problem: the agitation worsened, and L. started to bite her tongue. Risperidone was then prescribed at 1mg but caused loss of tonus and falls; lower doses of risperidone provided no clinical improvement and were discontinued.

### Clinical assessment

Clinical features were explored and a quantitative assessment was conducted in our outpatient clinic when the child was 7 years of age, and this evidenced poor developmental and adaptive skills. Behavioural observations were conducted during a 12-day stay in a free play setting with 5 other children, and through individualized assessments using the Psycho-Educative Profile and the Brunet-Lezine Scales. Scoring of the Child Autism Rating Scale was performed jointly by a psychiatrist, a psychologist, and a nurse at the end of the 12-day period of observation.

In the area of social interaction: L. was unable to point to indicate her needs, although she had been able to do it in the past according to her mother. She was able to make eye contact, but only for a few seconds, mostly on her own initiative. She expressed very few facial emotions, and she communicated by screaming. She was not interested in other children, but did not avoid them, whereas she liked to be close to adults. She was not interested in developing peer relationships.

In the area of communication: Her language was limited to a few words (yes, no, water), and she was not able to compensate by alternative modes of communication such as gesture or mime. She could not follow verbal instructions, nor imitate. She did not show interest in dolls, nor in pretend or symbolic play.

In the area of behaviours, interests and activities: We noted a short attention span, and rapid shifts from one uncompleted activity to another. She mainly engaged in low-grade repetitive activities, such as easy nesting games or playing with water. Incessant restlessness associated with clumsiness made her bump into objects or people and endanger herself because she failed to anticipate the consequences of her movements. She reacted to minor changes in her environment or to transitions from one activity to another by a worsening of her agitation, and induced vomiting. We observed pervasive eating disorders, including merycism, pica, and induced vomiting. L. would push objects deep inside her mouth or nose, keep her vomit in her mouth and swallow it again, causing disgust and rejection. However, she never showed any aggressive behaviour, towards herself or others, and had neither stereotyped movements nor rituals of persistent preoccupation with parts of objects.

On the Autism Diagnostic Interview- Revised (ADI-R) [19], she scored in the domain of reciprocal social interaction (20 > 10), communication (13 > 7), but not in the area of repetitive behaviours and stereotyped patterns (2 < 3) (Table 1). The total Child Autism Rating Scale score (CARS) [20] was 44. 5 (severely autistic) (Table 2).

**Table 1:** Autistic diagnostic interview-revised (ADI-R) scores

ADI-R domains	Scores
Reciprocal social interactions	**20**
-Failure to use nonverbal behaviors to regulate social interaction	2
-Failure to develop peer relationships	7
-Lack of shared enjoyment	4
-Lack of socio-emotional reciprocity	7
Language and Communication	**13**
-Lack of, or delay in, spoken language and failure to compensate through gestures	7
-Lack of varied spontaneous make-believe play	6
Restrictive, Repetitive and StereotypedBehaviors and Interests	**2**
-Encompassingpreoccupation or circumscribed pattern of interest	0
-Apparently compulsive adherences to nonfunctional routines or rituals	0
-Stereotyped and repetitive motor mannerisms	1
-Preoccupations with part-objects or non-functional elements of materials	1

**Table 2:** Child autism rating scale scores

CARS Items	Score
Imitation	4
Taste–smell-touch response	4
Verbal communication	4
Emotional response	3,5
Object use relationship to people	3,5
Fear and nervousness	3,5
General Impression	3,5
Body use	3
Adaptation to change	3
Non-verbal communication	3
Activity level	3
Relations to others and visual response	2,5
Listening response	2
Level and consistency of intellectual response	2
Global score	44,5

It was not possible to obtain a developmental profile with the Psycho-Educative Profile (PEP-R) [21] because of her agitation and lack of participation. On the Brunet-Lézine Scale [22], her developmental profile was heterogeneous, with developmental ages ranging from 14 months on language and socialization items, to 18 months on visuo-manual prehension coordination, and 42 months for global motor development. Neuropsychomotor assessment using standardized batteries [23] revealed impairments and delays for global motor development, visuo-manual prehension coordination, posturo-motor and locomotion acquisitions (static and dynamic balance). She was unable to draw a stick figure or a circle.

### Evolution

The agitation and gastrointestinal symptoms got worse. Therefore, we reintroduced treatment with risperidone 1 mg per day, with no effect. Risperidone was discontinued, and cyamemazine was introduced at low doses (10 mg), but her state did not improve. Her mother described the occurrence of 3 new episodes associating extreme fear and paleness without fever, separated by a few months, leading the neuropaediatrician to introduce a treatment with carbamazepine, despite a normal EEG. L. was then hospitalized for treatment adjustment in a full-time hospitalization unit. Digestive investigations showed gastro-oesophageal reflux, treated with omeprazole. The induced vomiting, merycism and pica improved. However, the agitation was still very great, and the regression in language and autonomy were persistent. By the time she was 10, she had completely lost her language abilities. She was extremely agitated, could not remain seated to eat. She still exhibited very frequent vomiting and merycism, would chew the sleeves of her pullover, pieces from jigsaw puzzles, and push them deep into her throat. She needed to wear diapers since she had completely lost toilet training, she would play with her dirty diapers and spread her faeces on the ground. She displayed sleep disorders, with difficulty falling asleep and frequent night-awakenings. The agitation, eating disorders and sleep problems were stabilized with continuation of a treatment associating risperidone (2mg), cyamemazine (25mg), carbamazepine (200mg), and omeprazole (10mg). However, her imitation and shared attention skills remained very limited. She continued to initiate the same repetitive activities, and paid little attention to other children.

### Physical assessment

Physical examination revealed no dysmorphic features. No auditory or visual impairment was found. The patient’s growth parameters were within the normal range.

### Neurophysiological investigations

EEG showed no seizure activity and there was no overt clinical seizure episode after the febrile seizure episodes at 5 years of age.

The cerebral magnetic resonance imaging (MRI) scan at 8 years of age showed a thin corpus callosum in the posterior part, hyper intensity on T2-weighted images localized in the hippocampus, and sub-cortical hyperintensity on T2-weighted images localized in the temporal lobes (bilateral temporal gliosis).

### Genetic investigations

**Fig. 1:**
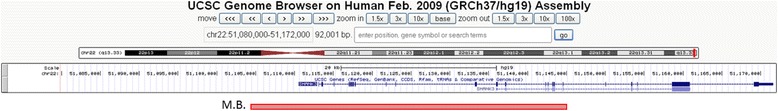
Representation of the 17 exons deletion of the SHANK3 gene

The patient had a first genetic screening which included karyotyping, screening for expansion of the CGG repeat sequence in the 5′ untranslated region of the *FMR1* gene and for mutations of the *MECP2* gene, using Sanger sequencing. This was followed up with general and metabolic screening (blood count and red cell parameters, blood urea, serum creatinine and electrolytes, liver function tests, thyroid function tests, levels of serum lactate, serum ammonia, serum lead, serum lactate and pyruvate, serum creatine and CPK, gas chromatography of blood and urine to screen for congenital metabolic disorders such as aminoacidaemia, organic acidaemia, and fatty acid oxidation disorders. No abnormality was found. We then performed an array comparative genomic hybridization (CGH) analyses which revealed a 22q13.3 deletion of 40, 8 kb (arr 22q13.3 (51,104,485-51,145,359)x1, hg19; NCBI build 37). Quantitative PCR (Polymerase Chain Reaction) and MLPA (Multiple Ligation Probe-dependent Amplification) of *SHANK3* gene confirmed the deletion. Data analyses indicated a deletion encompassing the first 17 exons of the *SHANK3* gene (Fig. 1). Only the mother was available for testing; she did not carry her daughter’s deletion.

## Discussion

We report the case of a girl carrying a 22q13.3 micro-deletion restricted to the first 17 exons of the *SHANK3* gene. The clinical features displayed by the girl strongly suggested the diagnosis of *dementia infantilis,* a developmental disorder described in 6 children by Heller in 1908 [24], characterized by (1) onset between 3 and 4 years of age after a period of apparently normal development, (2) a severe regression with the progressive loss or marked impairment of spoken language, loss of play, loss of social skills, and loss of bowel and bladder control, (3) a prodromal period of behavioural disruption with extreme agitation, fearfulness and (possibly) hallucinations and (4), a poor outcome with severe intellectual deterioration.

Indeed, one of the most important features presented by this case is the regressive pattern of her symptoms, with the progressive loss after the age of 3 years of her developmental and socialization skills, leading to severe adaptive impairment at 10 years. This developmental regression concerned not only the progressive loss of cognitive abilities such as language, socialization and attention, but also the loss of previously mastered automated functions: loss of toilet-training, pica, induced vomiting, merycism and sleep disorders. Furthermore, the regression was preceded in our patient by a prodromal phase characterized by behavioural disruption (restlessness and excitement, extreme agitation, short attention span with rapid shifts from one uncompleted activity to another), and the occurrence of acute episodes of extreme fearfulness associated with motor and neuro-vegetative symptoms, sometimes in a context of fever. The state of our patient progressively worsened after each episode. According to Westphal et al. [25], these episodes of extreme fear preceding a developmental regression are specific to Heller’s syndrome.

These acute episodes were first interpreted as partial epileptic frontal seizures. However, no abnormality was recorded on waking EEG in our patient, but we could not obtain a resting EEG because of her agitation. Interestingly, Figura et al. [10], in 6 patients with 22q13.3 deletion, reported a particular EEG pattern, characterized by multifocal paroxysmal EEG abnormalities, predominant over the frontal-temporal regions, activated during sleep.

*Dementia infantilis* was included in DSM IV under the heading *childhood disintegrative disorder* (CDD), with less stringent diagnostic criteria, especially with regard to age at onset (up to the age of 10 years). Our patient fulfilled the diagnostic criteria for CDD, in particular given the period of the first 2 years of normal development (walking at 12 months, first words at 10 months, first associations at 18 months and toilet-training by day and night around 2 years of age). The later regression of expressive and receptive language, social skills, bowel and bladder control, play and motor skills after the age of three, leading to qualitative impairment in social interaction, qualitative impairment in communication, and restricted, repetitive, and stereotyped patterns of behaviour indeed contribute to the definition of CDD.

The clinical case shown here could lead to several differential diagnoses. We eliminated the diagnosis of autistic disorder because L. did not have ritualized patterns of nonverbal behaviour. Criteria for Rett’s syndrome were not fulfilled since L. did not show a deceleration of head growth or stereotyped hand movements, and did not have mutations in the *MECP2* gene. Pervasive developmental disorder not otherwise specified is not a candidate diagnosis either since this category should be used when criteria are not met for a specific pervasive developmental disorder, like Childhood Disintegrative Disorder. Intellectual deficiency was associated with the diagnosis but could not explain the regressive pattern of the symptomatology, nor the presence of significant impairment in social functioning.

*Childhood disintegrative disorder* is extremely rare with an incidence of 1.7 in 100,000 children [26]. So far, very little is known on the genetic or metabolic factors associated with this specific condition, among which vitamin B12 deficiency [27] or anti-NMDA-receptor encephalitis [28]. Interestingly, *SHANK3* is a post-synaptic density protein involved in N-methyl-d-aspartate (NMDA) receptor tethering and dendritic spine rearrangement [29].

In addition, our case shares the same clinical features as other *SHANK3* deletion cases described in the literature: severe language impairment with impairment of social interactions, impairment of verbal and nonverbal communication, presence of sensory processing dysfunction involving touching and oro-facial activities, developmental delay and intellectual deficiency, hyperactivity and attention disorder, gastrointestinal symptoms and thin corpus callosum on cerebral MRI [3, 30].

Interestingly, severe regression in both language and social skills and adaptive functions (loss of motor skills, loss of independent eating and dressing, anorexia, loss of weight and urinary incontinence) have previously been reported in patients at different stages in development with a partial deletion or a single mutation of *SHANK3* gene [3, 9]: during childhood [11, 12, 31], in adolescence [1, 15, 16, 32] or in adulthood [17, 18].

Our case report confirms that regression may be part of the clinical phenotype and is perhaps a hallmark symptom of *SHANK3* haploinsufficiency, particularly in cases with a partial deletion or a single mutation.

### Limitations

One limitation to this observation is related to the fact that there was no home video to check that the development of L. was normal prior to the age of 2. However, the information in the mandatory child health data completed by the paediatricians confirmed that L.’s development was normal during the two first years of life. Another limitation is the fact that we could not obtain DNA from the father. However, we are confident about the pathogenicity of the deletion we describe, because deletions similar in position and size have already been described in patients: - Denayer et al. [17] reported a deletion of *SHANK3* encompassing the first 19 exons associated with bipolar disorder with progressive loss of skills in one of their 7 patients (patient 1); Bonaglia et al. [9] among their 44 patients, described two patients presenting small deletions encompassing the first 17 exons (patient P 37) and the first 9 exons (patient P44) respectively of the *SHANK3* gene, associated with Phelan-McDermid syndrome; patient P37 also exhibited a progressive loss of skills [10]. Finally, several cases of regression in language and behaviour after the age of 2 have been described in association with *SHANK3* mutations [11, 12, 31].

## Conclusion

In conclusion, we describe for the first time a partial deletion of the *SHANK3* gene associated with a clinical profile of childhood disintegrative disorder or Heller’s syndrome. We think it is crucial to carefully describe the timing of the appearance of autistic features in children, and to search for *SHANK3* mutations in patients diagnosed with childhood disintegrative disorder or any developmental disorder with a regressive pattern involving social and communicative skills as well as cognitive and automated functions, with onset around 3 years. Indeed, genetic diagnosis of *SHANK3* haploinsufficiency is important for both genetic counselling and care monitoring.

## Consent

Written informed consent was obtained from the patient for publication of this case report and any accompanying images. A copy of the written consent is available for review by the Editor of this journal.

## Abbreviations

ASD: Autism spectrum disorder
CDD: Childhood disintegrative disorder
PDD: Pervasive developmental disorder
EEG: Electroencephalogram
MRI: Magnetic resonance imaging
CGH: Comparative genome hybridization
NMDA: N-methyl-D-aspartate
